# Identifying predictors of cognitive decline in long-term care: a scoping review

**DOI:** 10.1186/s12877-023-04193-6

**Published:** 2023-09-05

**Authors:** Gaurav Arora, Christina Milani, Peter Tanuseputro, Patrick Tang, Ahwon Jeong, Daniel Kobewka, Colleen Webber

**Affiliations:** 1 University of Ottawa, Ottawa, ON Canada; 2 Ottawa Hospital Research Institute, Ottawa, ON Canada; 3 Western University, Windsor, ON Canada

**Keywords:** Long-term care, Nursing homes, Assisted-living, Cognitive decline, Cognitive impairment, Cognitive dysfunction, Risk factors, Protective factors, Associative factors

## Abstract

**Background:**

Cognitive impairment can cause social, emotional, and financial burdens on individuals, caregivers, and healthcare providers. This is especially important in settings such as long-term care (LTC) homes which largely consist of vulnerable older adults. Thus, the objective of this study is to review and summarize current research examining risk factors of cognitive decline in older adults within LTC.

**Methods:**

This scoping review includes primary observational research studies assessing within-person change in cognition over time in LTC or equivalent settings in high resource countries. A mean participant age of ≥ 65 years was required. Searches were conducted in Medline, Embase, Cumulative Index to Nursing and Allied Health Literature (CINAHL), and PyscInfo on June 27th, 2022 and included articles published during or after the year 2000. Title, abstract, and full-text screening was performed by two independent reviewers using Covidence. Specific predictors along with their associated relation with cognitive decline were extracted by a team of reviewers into a spreadsheet.

**Results:**

Thirty-eight studies were included in this review. The mean sample size was 14 620. Eighty-seven unique predictors were examined in relation to cognitive decline. Dementia was the most studied predictor (examined by 9 of 38 studies), and the most conclusive, with eight of those studies identifying it as a risk factor for cognitive decline. Other predictors that were identified as risk factors included arterial stiffness (identified by 2 of 2 studies), physical frailty (2 of 2 studies), sub-syndromal delirium (2 of 2 studies), and undergoing the first wave of COVID-19 lockdowns (2 of 2 studies). ADL independence was the most conclusive protective factor (3 of 4 studies), followed by social engagement (2 of 3 studies). Many remaining predictors showed no association and/or conflicting results.

**Conclusions:**

Dementia was the most common risk factor, while ADL independence was the most common protective factor associated with cognitive decline in LTC residents. This information can be used to stratify residents by risk severity and provide better personalized care for older adults through the targeted management of cognitive decline.

**Supplementary Information:**

The online version contains supplementary material available at 10.1186/s12877-023-04193-6.

## Background

Cognitive decline is characterized by increasing deficits in memory, thinking, and/or judgement [1]. Normal aging can involve gradual declines in cognitive abilities such as conceptual reasoning, memory, and processing speed [1]. However, some individuals may experience increasingly severe decline involving moderate to significant deterioration in one or more cognitive domains including complex attention, executive function, learning, language, perceptual-motor, and social cognition. This form of cognitive impairment may also contribute to a loss of independence when completing instrumental activities of daily living (IADLs), and when severe, can result in dementia, affecting basic activities of daily living (ADLs) [2].

Cognitive impairment can cause substantial social, emotional, and financial burdens on individuals, caregivers, and healthcare providers. As such, it is crucial to understand risk factors for cognitive decline before progression to inform care planning and risk mitigation efforts. This is especially important in settings such as long-term care (LTC) homes (also known as nursing homes), which provide nursing and personal care for some of the most vulnerable older adults, many of whom are already cognitively impaired. The risk of further cognitive decline among LTC residents is elevated due to older age, complex health needs and high degrees of frailty. Entry into LTC is an inflection point at which goals of care discussions are being had between residents, families and healthcare providers. Evidence on risk factors associated with cognitive decline can help inform these future care planning efforts and allow residents and families to prepare for future health outcomes. Moreover, identifying potentially modifiable risk factors in a timely fashion will provide opportunities to intervene to slow the risk and/or speed of decline.

While research has evaluated specific predictors of cognitive decline among LTC residents, there is a lack of review articles which synthesize the evidence. Therefore, the objective of this scoping review was to review and summarize research that examined risk factors of cognitive decline in older adults within LTC. Specifically, this review maps out the scope of the literature, describes the different types of characteristics that have been studied in relation to cognitive change, and identifies gaps in the literature.

## Methods

The protocol for this scoping review was prepared in accordance with the scoping review methodological framework originally developed by Arksey and O’Malley [3] and subsequently expanded by Levac et al. [4]. The scoping review protocol is available upon request from the corresponding author. This scoping review is reported in accordance with the guidelines described in the Preferred Reporting Items for Systematic Reviews and Meta-Analyses extension for Scoping Reviews (PRISMA-ScR).

### Eligibility criteria

To be included in this scoping review, studies had to meet all of the following inclusion criteria: studies measuring within-person change in cognition in relation to one or more risk factors; study population including residents of long-term care homes or equivalent facilities (i.e. nursing homes, elderly care homes); a mean participant age at the start of follow-up of ≥ 65 years; published during or after the year 2000 to reduce any potential differences in LTC residents and changes in care provision from pre-2000 to current times; and conducted in high resource countries to reduce differences in resident populations brought upon by substantially different healthcare systems including LTC – this was established by including countries that were defined as advanced economies as per the International Monetary Fund [5]. We excluded studies that used a cross-sectional design as they did not evaluate within-person change in cognition. We also excluded interventional studies as our intention was not to identify specific interventions that impact cognitive decline in LTC residents, but rather what factors, occurring outside of the context of medical care aimed at mitigating cognitive decline, may impact the risk of cognitive decline. Additionally, we also excluded conference abstracts, case reports, reviews, studies without full-text availability, and studies not published in English.

### Search strategy

The search strategy for this scoping review used Medline as the primary database, followed by a translation to Embase, the Cumulative Index to Nursing and Allied Health Literature (CINAHL), and PsycInfo. To ensure completeness of this search strategy, a health sciences librarian was involved in creating a comprehensive search methodology including appropriate MeSH terms, Boolean operators, and keywords. The Medline search strategy was then translated and applied to equivalent Embase, CINAHL, and PsycInfo terms. The search strategy is reported in Additional file 1. This scoping review also involved a grey literature search on search engines including Google and Bing, databases including OpenGrey, and targeted website searches including organizations like the Canadian Institute of Health Information, National Institute of Aging, and National Health Service. A combination of search terms was used to investigate the grey literature, each derived from the initial terms within the Medline search. These searches were initially conducted on July 26th, 2021, and then re-run on June 27th, 2022 to include newer publications.

### Selection of sources of evidence

After applying the search strategy to each database, the search results were exported into Covidence [6] and duplicates removed. We applied a two-step screening process, first evaluating citation titles and abstracts, and second evaluating the full texts of articles. During each step, two reviewers independently screened each record based on the aforementioned inclusion and exclusion criteria. If any disagreement for an article occurred, a meeting was held to discuss with a third and fourth reviewer to reach consensus. In regard to the grey literature search, no articles fell within this review’s eligibility criteria and thus no grey literature was included.

### Data charting process

Data extraction was completed using a Google Sheets file (Additional file 1). Prior to final extraction, a team of reviewers pilot tested the extraction sheet with five studies to ensure consistency in our process. Moving forward, all studies that were deemed to meet the study eligibility after full-text screening were independently extracted by two reviewers. Upon completion, the extracted data was compared for each study with any differences being discussed amongst all reviewers until agreement was achieved. We extracted data on study characteristics (author, publication year, country), study objectives, study population, inclusion and exclusion criteria, baseline age, sex, and presence of dementia, study recruitment period, duration of follow-up, variables analyzed as potential predictors of cognitive decline, tool(s) used to measure cognition, cognitive status at baseline, frequency of cognitive assessment during follow-up, administrator of cognitive assessment, definition of change in cognition, statistical approach, type of analysis used to evaluate predictors, and study results of associations between potential predictors and change in cognition, including whether the study identified that a predictor was a risk or protective factor for cognitive decline or not statistically significantly associated with cognitive decline. If a study reported results from both bivariate and multivariable analyses, we extracted results from only the multivariable analyses. Since the intent of this scoping review was to understand the current literature examining cognitive decline, no critical appraisal was completed on the articles included in the study.

### Data synthesis

Study characteristics were summarized using means, standard deviations, frequencies, and percentages. We grouped the predictors of decline that were evaluated in the reviewed studies into four thematic clusters: medical factors, functional and behavioural factors, medications, and demographic information. We described the number of potential predictors that were assessed in relation to cognitive decline across all the reviewed studies. For each potential predictor, we described the number of studies in which it was assessed, and of those studies, how many identified it as a risk factor and/or protective factor for decline, or not associated with decline. Because some studies evaluated predictors across different patient subgroups, it was possible for a single study to identify a potential predictor as both a risk factor and protective factor. In these studies, potential predictors were only characterized as being not associated with decline if the results were not statistically significant across all subgroups. We separately described the results for predictors that were examined by only one versus more than one study. Organizing predictor information in this manner allowed for the depiction of general trends as well as highlighting consistencies and inconsistencies across studies.

## Results

**Fig. 1:**
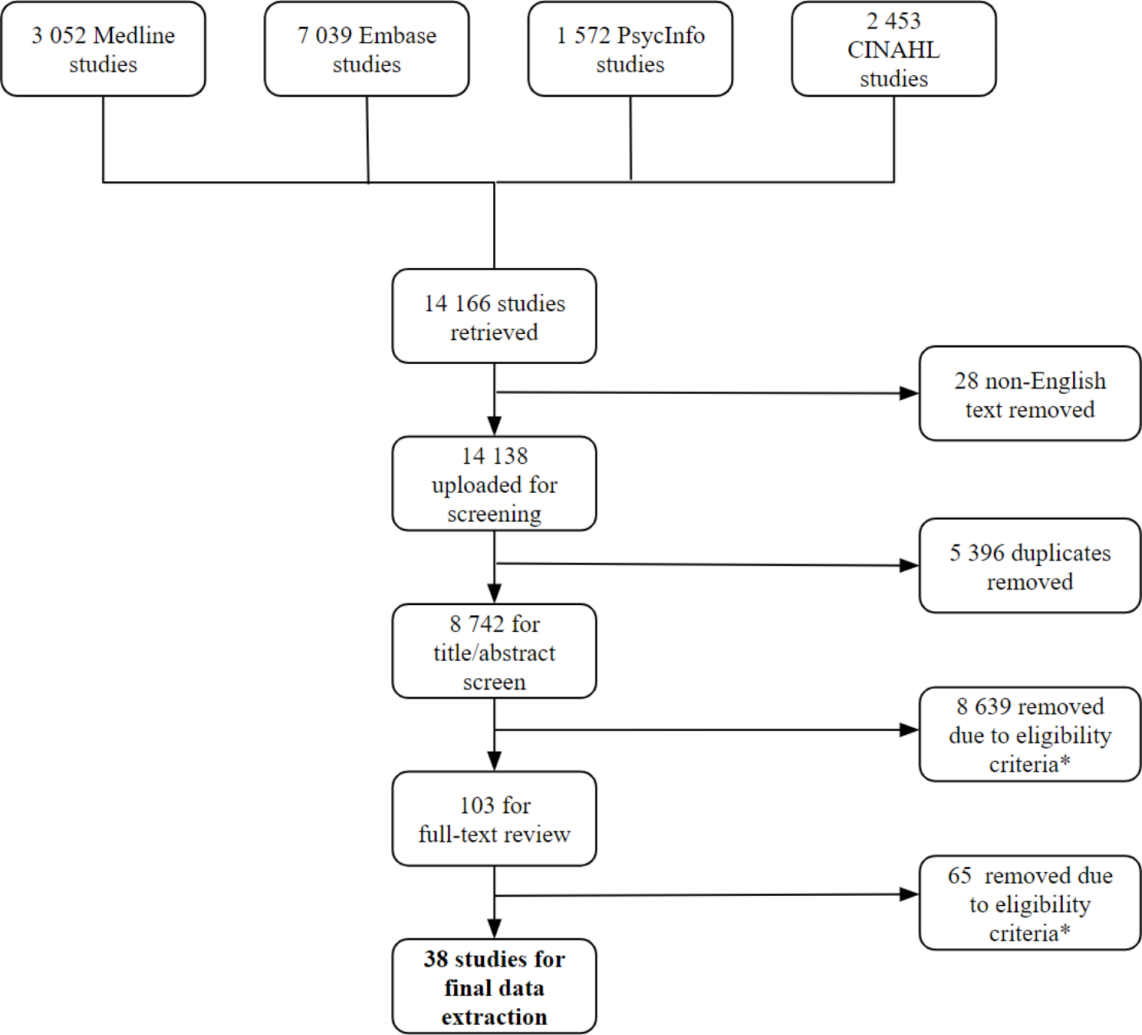
Selection Process Flowchart * Ineligible studies were excluded due to duplications, incorrect outcomes, settings, populations, lack of full-text availability, or ineligible study designs as per the selection criteria

Our database search yielded a total of 14 166 citations (Fig. 1). After removing duplicate studies (n = 5 396) and those not published in English (n = 28), 8 742 articles remained for screening. Following title and abstract screening, 103 articles met initial inclusion, and 38 remained after full-text screening.

### Study characteristics

The 38 included studies were published between 2002 and 2022. The majority of studies were conducted in the USA (n = 16) and Italy (n = 5). The sample size ranged from 26 to 266 001, with a mean sample size of 14 620 and a median of 771. Across the included studies, the mean age of study participants’ ranged from 65 to 93 years, the mean proportion of female participants was 66.5% and the mean proportion of participants who had dementia at baseline was 47.3% (Additional file 1).

### Measuring cognitive decline

The most common tool used to measure cognition was the Mini Mental State Examination (MMSE, used by 16 studies) followed by the Cognitive Performance Scale (CPS, used by 13 studies). Follow-up cognitive assessments were most commonly done on a quarterly basis, followed by annually. Follow-up time between measurements of cognition ranged from 3 months to 5.5 years. Cognitive decline was most commonly evaluated as the change in cognition score from baseline to end of follow-up. Others also used a dichotomous measure of cognitive decline, such as a ≥ 1 point increase in CPS or 3-point decrease in MMSE (Additional file 1).

### Predictors of cognitive decline assessed by > 1 study

Overall, 38 predictors were examined by > 1 study (Table 1). Medical factors were the most commonly studied group of variables, with 20 predictors being assessed in this category, followed by 10 in the functional and behavioural factors subgroup, and 4 each in the medications and demographics subgroups. Within the medical factors subgroup, dementia was the most frequently studied predictor of cognitive decline in LTC, being assessed by 9 different studies [7–15] with 8 out of 9 studies classifying it as a risk factor for decline [7, 8, 10−15] and the remaining 1 showing no association [9]. Eight of these studies used multivariable analysis to assess the relationship between dementia and subsequent cognitive decline, [7, 8, 10−15] over an aggregate sample of 299 469 [7–15]. Other predictors that showed an increased risk of cognitive decline included arterial stiffness (2 of 2 studies showing risk) [16, 17], physical frailty (2 of 2 studies showing risk) [15, 18], sub-syndromal delirium (2 of 2 studies showing risk) [11, 19], and COVID-19-related lockdowns (2 of 2 studies showing risk) [12, 20].

**Table 1:** Predictors Assessed by Multiple Studies

	Predictor of cognitive decline	# of studies assessing predictor	References	Cumulative sample size	# of studies with predictor as a risk factor	# of studies showing no association with predictor	# of studies with predictor as a protective factor	# of studies using multivariate analysis
**Cluster 1: Comorbidities**	Dementia	9	[7–15]	299 469	8	1	0	8
	Depression†	8	[7, 8, 10–15]	299 436	3	5	1	5
	Hypertension	5	[7, 15–17, 23]	269 084	1	4	0	4
	Full-syndromal delirium	4	[7, 9, 11, 24]	1 719	1	3	0	3
	Pain	4	[7, 9, 15, 25]	323 832	0	4	0	2
	Sensory impairment	4	[7, 8, 22, 31]	6 805	2	2	0	3
	Chronic lung disease	3	[7, 8, 15]	268 584	0	3	0	3
	Diabetes†	3	[7, 15, 32]	267 647	1	2	1	3
	Parkinsonism	3	[7, 8, 15]	268 584	0	3	0	1
	SARS-CoV-2 infection	3	[20, 26, 27]	673	0	3	0	2
	# of health conditions	3	[7, 9, 18]	10 984	0	3	0	2
	Arthritis	2	[7, 15]	267 305	0	2	0	1
	Cancer	2	[8, 15]	267 280	0	2	0	2
	Depression with dementia	2	[13, 14]	454	1	1	0	1
	Elevated arterial stiffness	2	[16, 17]	1 555	2	0	0	2
	Incontinence	2	[7, 8]	2 583	1	1	0	1
	Physical frailty	2	[15, 18]	275 648	2	0	0	2
	Pressure ulcers	2	[7, 8]	2 583	0	2	0	1
	Stroke	2	[7, 15]	267 305	1	1	0	2
	Subsyndromal Delirium	2	[11, 19]	350	2	0	0	2
**Cluster 2: Functional and Behavioural**	ADL independence	4	[7–10]	32 670	0	1	3	3
	Physical restraints	4	[7, 21, 33]	114 167	2	2	0	3
	Behavioural problems	3	[7, 8, 28]	19 387	1	2	0	1
	Social engagement	3	[8, 21, 22]	114 320	0	1	2	2
	Aggression	2	[7, 34]	1 330	1	1	0	0
	BMI	2	[7, 8]	2 583	0	2	0	0
	COVID-19 lockdown	2	[12, 20]	533	2	0	0	2
	Falls	2	[7, 8]	2 583	0	1	1	1
	Malnutrition	2	[8, 35]	2 527	0	1	1	1
	Weight loss	2	[7, 9]	1 337	0	2	0	0
**Cluster 3: Medications**	Antipsychotics	6	[7, 8, 15, 21, 33, 36]	399 118	2	3	1	4
	Antidepressants	4	[7, 15, 36, 37]	287 336	1	1	2	4
	Polypharmacy	3	[7, 9, 29]	5 961	0	3	0	2
	BZD/Z drugs	2	[15, 30]	266 227	0	2	0	2
**Cluster 4: Demographic Information**	Older age†	6	[7, 8, 10, 15, 18, 38]	308 972	4	2	2	5
	Female Gender†	3	[7, 10, 15]	297 359	1	1	2	3
	Marital status	2	[7, 10]	31 358	0	2	0	1
	Racial minority	2	[7, 15]	267 305	1	1	0	1

†These predictors were assessed by a study that classified the predictor as both risk and protective factors of cognitive decline. Thus, the “# of studies assessing predictor” column is less than the sum of the columns showing # of studies as a risk factor, protective factor, or no association

Increased ADL independence was a protective factor in 3 of 4 studies [7, 8, 10] and the remaining study found no association with decline [9]. Three of these studies used multivariable analysis [7, 8, 10] over an aggregate sample of 32 670 [7–10]. Increased social engagement was a protective factor in 2 of 3 studies [21, 22] and the remaining study found no association with cognitive decline [8].

For 17 predictors that were evaluated by > 1 study, the majority (> 50%) of studies indicated that they had no significant association with cognitive decline. These include depression (5 of 8 studies reporting no association with cognitive decline) [7, 8, 10−15], hypertension (4 of 5 studies) [7, 15–17, 23], full-syndrome delirium (3 of 4 studies) [7, 9, 11, 24], pain (4 of 4 studies) [7, 9, 15, 25], chronic lung disease (3 of 3 studies) [7, 8, 15], Parkinsonism (3 of 3 studies) [7, 8, 15], SARS-CoV-2 infection (3 of 3 studies) [20, 26, 27], number of health conditions (3 of 3 studies) [7, 9, 18], arthritis (2 of 2 studies) [7, 15], cancer (2 of 2 studies) [8, 15], pressure ulcers (2 of 2 studies) [7, 8], behavioural problems (2 of 3 studies) [7, 8, 28], body mass index (2 of 2 studies) [7, 8], weight loss (2 of 2 studies) [7, 9], polypharmacy (3 of 3 studies) [7, 9, 29], benzodiazepines and Z-drug use (2 of 2 studies) [15, 30], and marital status (2 of 2 studies) [7, 10]. In addition, 14 predictors had inconclusive findings, whereby no more than 50% of the studies in which they were evaluated identified them as a risk factor, protective factor, or not associated with cognitive decline. Inconclusive predictors include sensory impairment [7, 8, 22, 31], diabetes [7, 15, 32], depression with dementia [13, 14], incontinence [7, 8], stroke [7, 15], physical restraints [7, 21, 33], aggression [7, 34], falls [7, 8], malnutrition [8, 35], antipsychotic use [7, 8, 15, 21, 33, 36], antidepressant use [7, 15, 36, 37], older age [7, 8, 10, 15, 18, 38], female gender [7, 10, 15], and being a racial minority [7, 15].

### Predictors of cognitive decline assessed by 1 study

Forty-nine predictors were assessed in relation to cognitive decline by only one study (Table 2). The majority of these predictors (38 of 49) were reported to have no significant association with cognitive decline [7–9, 13, 15, 17, 18, 36, 39−43]. Of the remaining predictors, nine were identified as risk factors for cognitive decline. These include epilepsy [15], hip fracture [15], hospitalization for infection [44], hearing aid use [31], living with others prior to nursing home admission [10], not living in a private household prior to admission [10], rural nursing home placement [15], increased number of Resident Assessment Protocol (RAP) triggers [8], and poor performance on the MMSE domain orientation for time [43]. Amongst each of these nine risk factors, all but one (hearing aid use) were assessed using multivariable analyses [8, 10, 15, 31, 43, 44]. Visual aid use [31] and anti-dementia medications [36] were the only protective factors and only anti-dementia medications were assessed using multivariable analysis [36].

**Table 2:** Predictors Assessed Once by an Individual Study

	Predictor of cognitive decline	References	Sample Size	Risk factor, protective factor, or no association	Use of multivariate analysis
**Cluster 1: Comorbidities**	Anxiety	[15]	266 001	No association	Yes
	Coronary artery disease	[7]	1 304	No association	No
	Epilepsy	[15]	266 001	Risk	Yes
	Heart failure	[15]	266 001	No association	Yes
	Hip fracture	[15]	266 001	Risk	Yes
	History of depression	[13]	313	No association	Yes
	Infection related hospitalization	[44]	20 698	Risk	Yes
	Medical instability	[9]	33	No association	No
	Multiple sclerosis	[15]	266 001	No association	Yes
	Osteoporosis	[15]	266 001	No association	Yes
	Pulse pressure amplification	[17]	682	No association	Yes
	Sarcopenia	[39]	58	No association	Yes
	Tachycardia	[17]	682	No association	Yes
**Cluster 2: Functional and Behavioural**	Decreased oral intake	[7]	1 304	No association	Yes
	Dysphagia	[7]	1 304	No association	Yes
	Hearing aid use	[31]	2 233	Risk	No
	Height	[8]	1 279	No association	No
	Institutionalized > 5 years	[8]	1 279	No association	No
	Living alone before admission	[7]	1 304	No association	Yes
	Living with others before admission	[10]	30 054	Risk	Yes
	Medicaid	[7]	1 304	No association	No
	Not living in a private household before admission	[10]	30 054	Risk	Yes
	RAP - communication	[8]	1 279	No association	No
	RAP - dental care	[8]	1 279	No association	No
	RAP - functional rehabilitation	[8]	1 279	No association	No
	Rural nursing home	[15]	266 001	Risk	Yes
	Suprapubic tube placement	[18]	9 647	No association	Yes
	Tube feeding	[7]	1 304	No association	Yes
	Unstable cognition	[7]	1 304	No association	No
	Visual aid use	[31]	2 233	Protective	No
	Weight	[8]	1 279	No association	No
**Cluster 3: Medications**	Anti-anxiety medications	[15]	266 001	No association	Yes
	Anticholinergics	[40]	3 536	No association	Yes
	Anti-dementia medications	[36]	18 950	Protective	Yes
	Estrogen use	[41]	854	No association	Yes
	Glucose lowering medications	[42]	1 784	No association	Yes
	Mood stabilizers	[36]	18 950	No association	Yes
**Cluster 4: Demographic Information**	Educational years	[8]	1 279	No association	No
**Cluster 5: Other**‡	Baseline MDS COGS	[8]	1 279	No association	No
	CPS score	[9]	33	No association	No
	DNR	[7]	1 304	No association	Yes
	Higher number of RAP triggers	[8]	1 279	Risk	Yes
	MDS COGS decline	[8]	1 279	No association	No
	MMSE Score	[8]	1 279	No association	No
	Poor performance on MMSE - orientation for time	[43]	505	Risk	Yes
	Poor performance on MMSE - delayed recall	[43]	505	No association	Yes
	Poor performance on MMSE - attention	[43]	505	No association	Yes
	Poor performance on MMSE - orientation for place	[43]	505	No association	Yes
	Presence of living will	[7]	1 304	No association	No

‡ Predictors falling under this cluster either encompassed more than one of the other clusters, or do not fit well into previous clusters

## Discussion

This scoping review aimed to summarize the current literature identifying factors that are associated with cognitive decline in elderly LTC residents. Ultimately, there were several key risk factors and protective factors identified among this population. Predictors that were evaluated in more than one study and identified as risk factors in > 50% of studies included dementia [7, 8, 10−15] arterial stiffness [16, 17], physical frailty [15, 18], sub-syndromal delirium [11, 19], and lockdowns for COVID-19 [20]. Predictors that were evaluated in more than one study and identified as protective factors in > 50% of studies included ADL independence [7, 8, 10] as well as social engagement [22]. Many predictors were assessed by only a single study and thus those findings may not be truly representative of the associated risk. Due to the difficulty in assessing the consistency of results across these individual studies, more research in this area is warranted.

The evidence generated by this scoping review has potential implications for LTC care planning and delivery as well as research. Among the predictors that had findings suggestive of being risk or protective factors for cognitive decline, dementia, sub-syndromal delirium, physical frailty, ADL independence, and social engagement are relatively easily identifiable conditions or states. They may thus serve as easy flags of LTC residents who may be in need of further cognitive screening, or rather, point toward those that may have a more favourable trajectory. By synthesizing the evidence on risk and protective factors for cognitive decline in LTC residents, the findings of this scoping review could also inform the development of risk prediction tools that can identify older adults entering LTC who may be at a lower or higher risk of cognitive decline. Such a tool may help healthcare providers with personalized care planning that involves communicating with family members to give them an idea of what to expect – particularly important given that LTC residents are often in their last months or years of life – and may shape the trajectory of their future plans.

Evidence of characteristics associated with the risk of cognitive decline in LTC residents may also help inform efforts to treat and mitigate underlying conditions in a timely manner. While the aim of this scoping review was not to identify factors that are causally related to cognitive decline, our findings may be used to generate potential hypotheses of causes of cognitive decline that warrant further study. For instance, while increased social engagement may simply be a marker for a different underlying process that is protecting against cognitive decline, it may alternatively protect against cognitive decline through causal processes [45]. Evidence of the latter may help inform the development of interventions to support healthy cognitive aging of LTC residents. Further research is needed to better understand the pathways through which cognitive impairment in LTC residents occurs.

As well, analysis of the subgroups of predictors reveals less study being done on the medication and demographics clusters, potentially hinting at an opportunity for further research. Specifically, gender and ethnicity represent important parameters to consider yet are poorly represented in the literature. Furthermore, many of the predictors examined by only one study may benefit from replication studies to create more robust trends. This may uncover new themes which were not identified in this review, such as factors relating to nursing homes themselves. Therefore, the findings uncovered in this review not only depict strong evidence for factors influencing cognitive decline, but also prompt further research of predictors that were unclear.

This scoping review has a number of strengths. Notably, this is the first scoping review to assess predictors of cognitive decline specifically within LTC and can pave the way toward future research in this field. Moreover, the robust search, screening and data extraction strategy used in this review allowed for a comprehensive inclusion of the most relevant literature. Our study has several limitations. First, as noted above, the predictors identified in this review cannot be interpreted as causally associated with cognitive decline without further research; as such, the conclusions and potential implications of this review must be made with that limitation in mind. Second, variations in measurement of cognition and analysis of cognitive decline likely contributed to inconsistent findings across studies. Differences in the sensitivity and specificity of cognitive assessments such as the MMSE and CPS may lead to different rates of false positives and false negatives in cognitive assessment. Further, certain tools, such as the CPS which has only 6 levels of cognition, may be less sensitive to changes in cognition than other tools such as the MMSE which uses a 30-point scale [46]. Moreover, the varying definition of change in cognition used by studies may further lead to differing results. Third, variations in how predictors were defined, measured, and categorized may have also contributed to inconsistent findings across studies. Fourth, we did not conduct any assessment of study quality or potential sources of bias to remain consistent with a scoping review methodology. Finally, while we used a comprehensive search strategy, we may have excluded relevant research if it was not published in English.

## Conclusions

In conclusion, this scoping review outlines the current literature regarding factors that have been studied in relation to cognitive decline among older adults in LTC. The findings identify several resident characteristics associated with cognitive decline, with pre-existing dementia and ADL independence being the most frequently studied risk and protective factors, respectively. Risk prediction models for cognitive decline, informed by this research, may be useful tools to incorporate into LTC practice in order to identify LTC residents who are at high risk of cognitive decline. A better understanding of the causes of cognitive decline may help with efforts to prevent cognitive decline and maintain cognition amongst LTC residents.

### Electronic supplementary material

Below is the link to the electronic supplementary material.

Supplementary Material 1: Additional file 1 is an excel document containing the Medline search strategy along with the data extraction table from which final results were derived.

## Data Availability

All data generated or analysed during this study are included in this published article and its supplementary information files.

## Abbreviations

LTC: long-term care
ADL: activities of daily living
IADL: instrumental activities of daily living
MMSE: Mini Mental State Examination
CPS: Cognitive Performance Scale
